# Treatment strategy for HER2-negative advanced gastric cancer: salvage-line strategy for advanced gastric cancer

**DOI:** 10.1007/s10147-024-02500-8

**Published:** 2024-05-11

**Authors:** Naohiro Nishida, Daisuke Sakai, Taroh Satoh

**Affiliations:** 1 Center for Cancer Genomics and Personalized Medicine, Osaka University Hospital, 2-2 Yamada-oka, Suita, Osaka 565‐0871 Japan; 2 Department of Medical Oncology, Osaka International Cancer Institute, Osaka, Japan

**Keywords:** Advanced gastric cancer, Later-line treatment, Immune checkpoint inhibitor (ICI), Anti-VEGF treatment, Tyrosine kinase inhibitors (TKIs)

## Abstract

After immune checkpoint inhibitor (ICI) comes into third-line treatment of advanced gastric cancer, the therapeutic strategy has been dramatically changed. Recent first-line regimen, which consists of ICI and chemotherapeutic agents, prolonged progression-free survival, and subsequent treatment options enabled continuous treatment beyond second-line therapy. Moreover, the advent of vascular endothelial growth factor (VEGF)-targeted agents including angiogenesis inhibitors and TKIs provides an opportunity of considering the interaction between ICI and anti-VEGF agents, and facilitating novel treatment proposal. Although clinical benefit of prolonged VEGF blockade after disease progression has not been confirmed in gastric cancer, combination therapy of cytotoxic agents and anti-VEGF agent, such as irinotecan plus ramucirumab demonstrated favorable objective response rate and progression-free survival in third- or later-line setting. In this review, we discuss recent progress and future directions of later-line treatments of HER2-negative advancer gastric cancer.

## Introduction

**Fig. 1:**
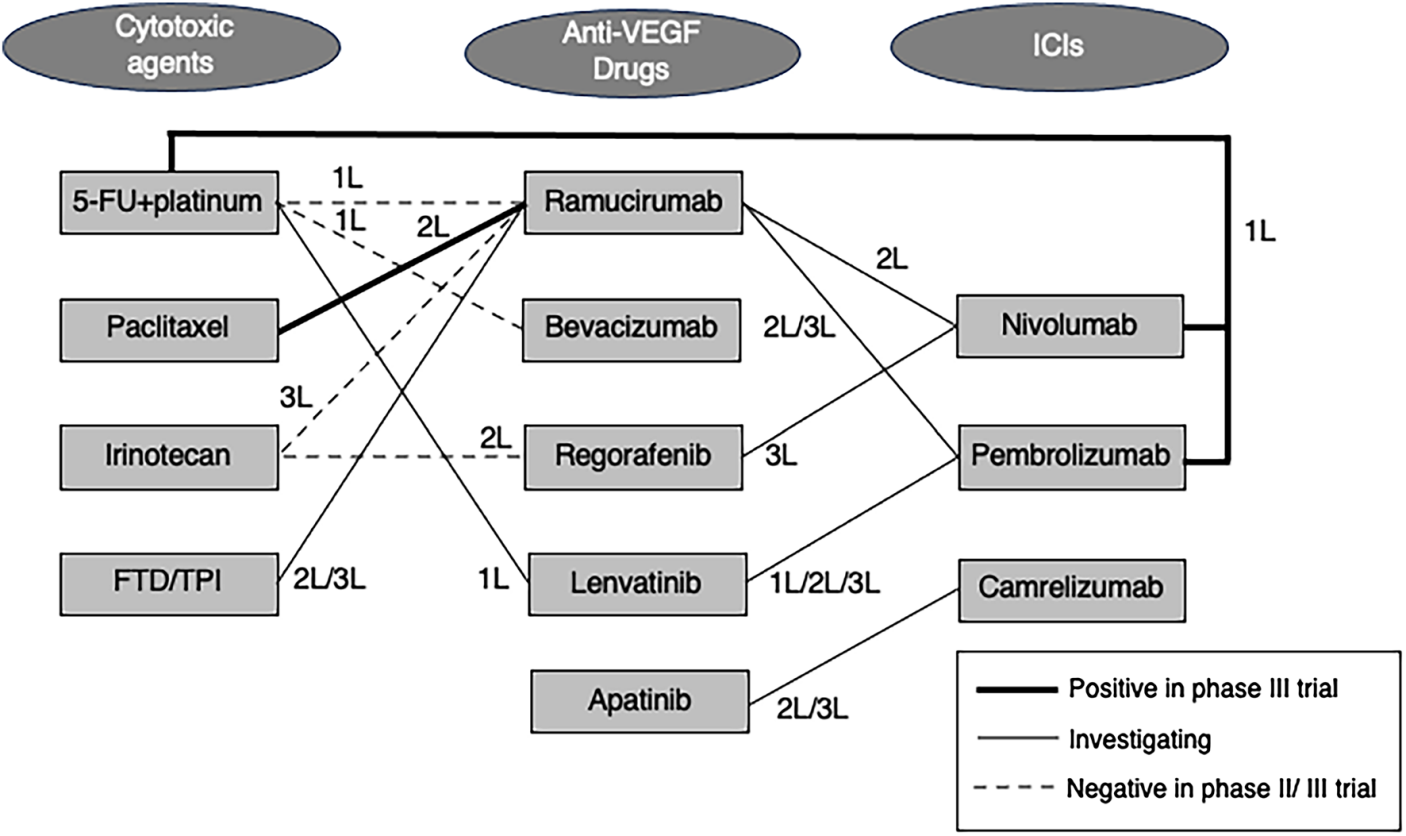
Combination therapy for advanced gastric cancer. Not all trials are included. *1L* first line, *2L* second line, *3L* third line

Because of fragility of patients who have undergone first- and secondary-line treatments, maintaining a performance status (PS) that allows for the continuation of ongoing treatment is one of the crucial factors in later-line treatment strategy of advanced gastric cancer. Stomach, being located upstream in the digestive tract, presents significant challenges in terms of nutritional intake in pathological condition. Particularly in tumors affecting the pyloric region, gastrointestinal obstructions can be a serious complication. Surgical interventions, including gastrojejunostomy, may be necessary, and a multidisciplinary team's involvement in palliative and nutritional care carries significant meaning [1]. Moreover, in gastric cancer, unlike other cancer types, there are instances where measurable lesions were not detectable on CT images, making it challenging to precisely determine the progression of the disease and timing to change treatment. To sustain treatment over an extended period, it is essential not only to conduct regular imaging diagnostics to promptly assess the progression of the disease but also to closely monitor tumor markers, prognostic indicators, and, above all, the patient's symptoms [2]. In this context, the advent of ICIs, which do not seriously affect general condition except for some immune-related adverse effects (irAEs), holds significant importance for fragile gastric cancer patients. Tumors in the upper gastrointestinal tract are known to have higher immunogenicity compared to those in the lower gastrointestinal tract, making them generally more responsive to ICIs [3]. Gastric cancer is commonly classified into molecular subtypes, including Epstein–Barr virus-positive (EBV), microsatellite instability (MSI), genomically stable (GS), and chromosomal instability (CIN) [4]. Not only in MSI and EBV subtype, which are known to have higher immunogenicity, but also in all these subtypes, it has been observed that CD8-positive T cell infiltration and IFN-gamma signaling are more pronounced compared to lower gastrointestinal tumors [3]. Furthermore, attention is being focused on interaction between anti-VEGF therapy and ICIs. These two drugs are molecularly targeted therapies that affect the tumor microenvironment rather than the tumor itself. It has been observed that the response rate of nivolumab in third-line treatment is higher in groups using ramucirumab in second-line treatment [5], and conversely, patients treated with nivolumab show increased sensitivity to taxanes and ramucirumab combination chemotherapy [6]. This suggests the potential modification of the tumor immune microenvironment by anti-angiogenic agents. Not only the extension of progression-free survival (PFS) based on the performance of drugs in each treatment line but also interactions among various drugs throughout the total course of treatment may contribute to the extension of overall survival. Figure 1 illustrates the correlation diagram of combination therapy for each drug along with treatment lines (Fig. 1). This review provides an overview of the roles of key drugs in the later-line treatment of HER2-negative gastric cancer and also discusses the interactions between each drug, aiming to facilitate a comprehensive understanding of the overall management of gastric cancer treatment.

## Angiogenesis inhibitors in later-line treatment

**Fig. 2:**
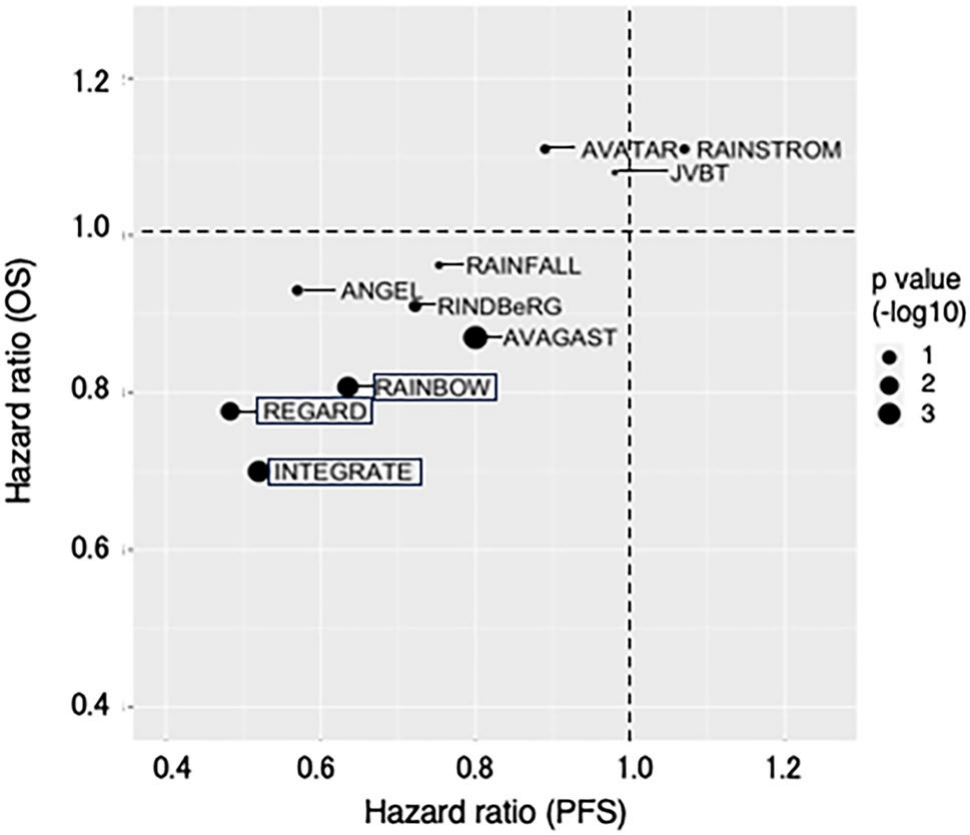
Scatter plot for hazard ratio of PFS and OS in clinical trials of anti-VEGF agents in gastric cancer. Size of the circle represents − log 10 *p* value of OS hazard ration. The trial names which showed prolonged OS were surrounded by square

In VEGF signaling pathway, there exist five ligands, namely, VEGF-A, VEGF-B, VEGF-C, VEGF-D, and ﻿placental growth factor (PlGF) along with three receptors, VEGFR-A, VEGFR-B, and VEGFR-C. Bevacizumab is a monoclonal antibody (mAb) against VEGFR-A ligand, while ramucirumab targets VEGFR2, which is expressed in vascular endothelial cells, normal epithelium, and tumor cells [7, 8]. The development of angiogenic inhibitors for gastric cancer treatment has not progressed as smoothly as in colorectal cancer. In AVAGAST trial, the addition of bevacizumab to platinum doublet chemotherapy increased ORR and improved PFS, but did not demonstrate a prolongation of OS in first-line treatment [9]. Similarly, multiple clinical trials, including phase III RAINFALL trial for ramucirumab, did not demonstrate prolonged OS by addition of ramucirumab to standard chemotherapy in first-line gastric cancer treatment [10]. There are three clinical trials, RAINBOW, REGARD, and INTEGRATE in which anti-VEGF therapy improved OS in second and later-line setting (Fig. 2) [11–13]. The REGARD trial, an international, multicenter phase III trial, evaluated the efficacy of ramucirumab against best supportive care (BSC) in secondary treatment for advanced gastric adenocarcinoma or gastroesophageal junction adenocarcinoma previously treated with fluoropyrimidine and platinum. Additionally, the RAINBOW trial in second-line treatment demonstrated an OS extension with combination therapy of ramucirumab and paclitaxel compared to paclitaxel alone. Apatinib, another anti-VEGF therapy, a highly selective VEGFR-2 inhibitor, showed significant OS and PFS benefits in the Chinese population, but these results were not confirmed in the global phase III, ANGEL trial [14]. There is still no clear evidence of OS improvement for the use of anti-angiogenic agents beyond progression in third-line treatment and beyond. On the other hand, the multikinase inhibitor, regorafenib, significantly prolonged OS and PFS compared to BSC in gastric cancer salvage-line treatment in the INTEGRATE trial [13]. There is enthusiasm for combination therapy of regorafenib and ICIs, which is discussed in the following section.

## Regorafenib

Regorafenib (BAY 73-4506) is a multikinase inhibitor that targets for angiogenic (VEGFR1, VEGFR2, VEGFR3), stromal (PDGFR), and other oncogenic (KIT, RET, and RAF) receptor tyrosine kinases and is widely used for the treatment of malignancies including colorectal cancer, gastrointestinal tumors (GISTs), and hepatocellular carcinoma. The phase III CORRECT trial comparing regorafenib monotherapy with placebo demonstrated improved overall survival in patients with previously treated metastatic colorectal cancer [15]. However, the efficacy of a multikinase inhibitor in later-line treatment of advanced gastric cancer has not been investigated. International randomized phase II trial, INTEGRATE, showed prolonged PFS in gastric cancer patients treated with regorafenib compared to placebo group [13]. Moreover, pooled analysis combining INTEGRATE (phase II trial) and INTEGRATE IIa (phase III trial) demonstrated that regorafenib improved OS (HR 0.70; 95% CI 0.56–0.87; *p* = 0.001) and PFS (HR 0.53; 95% CI 0.40–0.70, *p* < 0.0001) [16]. Prior use of VEGF inhibitors does not significantly affect the prognosis in subgroup analysis. Regorafenib was also shown to delay a deterioration of quality of life (QOL). Because of relatively high toxicity including fatigue, hand–foot syndrome, liver dysfunction, and hypertension of this drug, patients frequently require dose reduction in clinical use. However, the results showed most of the adverse effects are manageable even in later-line gastric cancer patients. Regorafenib is also promising drug to enhance the antitumor activity of ICIs. In vitro models have demonstrated that anti-VEGF drugs including regorafenib and ramucirumab promote infiltration of CD8 T cell and simultaneously inhibit effector regulatory T cells (eTreg cells), which is a favorable microenvironment for immune-therapy [17, 18]. According to this concept, phase Ib study of regorafenib plus nivolumab was conducted on gastric and colorectal cancer who have received at least two chemotherapies. The results showed relatively high overall response rate, 44% (95% CI 24.4–65.1%) in gastric cancer patients and 36% (95% CI 18.0–57.5%) in colorectal cancer patients [19]. INTEGRATE IIb, a randomized phase III trial of regorafenib plus nivolumab comparing to standard chemotherapy, is ongoing in pretreated advanced gastroesophageal cancer [20]. Recently, efficacy of regorafenib in combination with nivolumab and chemotherapy showed promising activity as first-line treatment in a single-arm phase II trial [21]. Although most of the adverse events are manageable, frequency of grade 3 or worse adverse events were relatively higher than that of combination of nivolumab and chemotherapy without regorafenib. Efficacy of this regimen in first-line treatment needs to be further investigated in phase III trial (Table 1).

**Table 1:** Clinical trials of anti-angiogenic agents with or without chemotherapy

Trial name	Anti-VEGF agents	Treatment line	Trial phase	mPFS (95% CI) (month)	Hazard ratio (PFS) (95% CI)	*p* value (PFS)	MST (95% CI) (month)	Hazard ratio (OS) (95% CI)	*p* value (OS)
AVAGAST	Bevacizumab	1	III	6.7 (5.9–7.1) vs 5.3 (4.4–5.6)	0.80 (0.68–0.93)	0.0037	12.1(11.1–13.8) vs 10.1 (9.0–11.3)	0.87 (0.73–1.03)	0.1002
AVATAR	Bevacizumab	1	III	6.0 (4.9–7.4) vs 6.3 (5.7–7.4)	0.89 (0.66–1.21)	0.47	11.4 (8.6–16.0) vs. 10.5 (8.9–14.1)	1.11 (0.79–1.56)	0.5567
RAINFALL	Ramucirumab	1	III	5.7 (5.5–6.5) vs 5.4 (4.5–5.7)	0.75 (0.61–0.94)	0.0106	11.2 (9.9–11.9) vs. 10.7 (9.5–11.9)	0.96 (0.80–1.16)	0.6757
RAINSTROM	Ramucirumab	1	rII*	6.3 vs. 6.7	1.07 (80% CI 0.86–1.33)	0.7	14.7 vs. 14.3	1.11 (80% CI 0.89–1.40)	0.55
JVBT	Ramucirumab	1	rII*	6.4 vs. 6.7	0.98 (95% CI 0.69–1.37)	0.886	11.7 vs. 11.5	1.08 (95% CI 0.73–1.58)	0.712
RAINBOW	Ramucirumab	2	III	4.4 (4.2–5.3) vs 2.9 (2.8–3.0)	0.64 (0.54–0.75)	< 0.001	9.6 (8.5–10.8) vs 7.4 (6.3–8.4)	0.81 (0.68–0.96)	0.017
REGARD	Ramucirumab	2	III	2.1 (IQR 5.9–7.1) vs 1.3 (IQR 1.3–4.2)	–	–	5.2 (IQR2.3–9.9) vs 3.8 (IQR 1.7–7.1)	0.78 (0.60–0.99)	0.047
APATINIB	Apatinib	≧ 2	III	2.83 vs 1.77	0.57 (0.46–0.79)	< 0.0001	5.78 vs 5.13	0.93 (0.74–1.15)	0.485
INTEGRATE	Regorafenib	≧ 2	III	1.8 v 1.6	–	< 0.0001	5.0 vs 4.1	0.69 (0.56–0.87)	0.001

Not all trials are included. *rII randomized phase II

## ICIs

First evidence showing efficacy of ICI in gastric cancer came from phase III ATTRACTION-2 trial, in which nivolumab monotherapy significantly improved overall survival compared to placebo (median overall OS 5.26 months in the nivolumab group and 4.14 months in the placebo group; HR 0.63; *p* < 0.0001) in patient refractory to standard therapy [22].

Importantly, duration of confirmed response was 9.53 month (95% CI 6.14–9.82) and overall survival rate in 12-month was 26.2% in nivolumab group, showing a long duration of response of an immunotherapy agent. Although this study opens up a new avenue for ICI in advanced gastric cancer, comparison of the efficacy between ICI and chemotherapy was not evaluated in this trial. Subsequent KEYNOTE-061 trial comparing pembrolizumab with paclitaxel in second-line setting did not show significant survival benefit of pembrolizumab monotherapy compared with paclitaxel [23]. However, pivotal trials including phase III CHECKMATE-649 and ATTRACTION-4 trial demonstrated an efficacy of combination therapy with ICI and chemotherapy in first-line treatment, making this combination therapy an essential treatment option in HER2-negative advanced gastric cancer. Reinduction of ICI in later-line treatment is not recommended in case ICI combined regimens are selected as first-line treatment [24]. On the other hand, nivolumab is frequently used as later-line treatment of HER2-positive gastric cancer, in which trastuzumab combined chemotherapy is a standard first-line therapy. Post hoc subgroup analysis in patients with prior trastuzumab use in ATTRACTION‑2 trial showed that patients with prior trastuzumab use, who were predicted to be HER2-positive cases with high probability, showed comparable, even better prognosis compared with patients who were not treated with trastuzumab [25], encouraging the induction of nivolumab as salvage-line treatment in HER2-positive gastric cancer. Interaction between ICI and anti-VEGF therapy are also indispensable factor in recent cancer treatment strategy. Subgroup analysis of ATTRACTION‑2 trial showed favorable objective response rate (ORR) and progression-free survival (PFS) in nivolumab group with prior ramucirumab use than in those without [5]. Moreover, improved efficacy of taxanes and ramucirumab combination chemotherapy after exposure to anti-PD-1 therapy is also observed [6], suggesting the immunomodulatory effects of anti-VEGF drugs.

## Irinotecan

Irinotecan is one of key drugs for various types of malignancies including gastric cancer. Although phase III study comparing irinotecan with paclitaxel after progression of first-line fluoropyrimidine and platinum therapy showed no significant difference on OS between two chemotherapeutic agents [26], addition of ramucirumab to paclitaxel significantly increased overall survival compared with paclitaxel alone, leading this combination therapy as new standard second-line treatment for advanced gastric cancer [11], and irinotecan is commonly used in later-line treatment as monotherapy. Other anti-VEGF monoclonal antibody, bevacizumab, is shown to have prognostic benefit even after the progression of prior combination therapy with bevacizumab, so called bevacizumab beyond progression in colorectal cancer [27]. Also, addition of ramucirumab to FOLFIRI significantly improved overall survival as second-line treatment for patients with metastatic colorectal cancer that progressed after first-line treatment with bevacizumab plus chemotherapy [28]. However, the efficacy of prolonged use of anti-VEGF agents including ramucirumab after disease progression has not been clearly demonstrated. RINDBeRG trial, phase III randomized controlled trial, comparing ramucirumab plus irinotecan with irinotecan in the third- or later-line treatment answered this question. Patients with advanced gastric cancer were recruited from over one hundred institutions consisting of 9 clinical trial groups in Japan. Primary endpoint is OS and secondary endpoints is progression-free survival, time to treatment failure, response rate, disease control rate, and safety. The primary end point was not met, and median OS was comparable between the combination of irinotecan plus ramucirumab and irinotecan alone (9.4 months vs 8.5 months, adjusted HR 0.91; *p* = 0.369). On the other hand, median PFS was favorable (3.8 months vs 2.8 months; HR 0.722; *p* = 0.001), and ORR was also higher in combination group (22.1% vs 15.0%) [29]. The result demonstrated that sustained VEGF blockade beyond progression does not bring clinical benefit in advanced gastric cancer, although addition of ramucirumab to irinotecan contributes to more tumor regression and prolonged PFS. The combined therapy with ramucirumab and irinotecan demonstrated acceptable safety profile, with no emergence of new or unexpected adverse effects observed in the trial. According to phase II trial investigating the clinical efficiency of ramucirumab plus irinotecan as second-line treatment, median PFS and OS were 4.2 months (95% CI 2.5–5.4 months) and 9.6 months (95% CI 6.4–16.6 months), respectively [30]. The results are similar to that of RINDBeRG trial, which is in third- or later-line setting, suggesting the efficacy of this regimen even in salvage-line treatment. The results of RINDBeRG trial suggest potential benefit of sustained VEGF inhibition by switching backbone chemotherapy.

## Trifluridine/tipiracil (FTD/TPI)

In the recent decade, most of the newly approved drugs are molecular-targeted agents, and pharmaceutical companies have not focus on drug development of cytotoxic agents. The exception is FTD/TPI, oral combination tablet of thymidine-based nucleoside analog (trifluridine) and potent thymidine phosphorylase inhibitor, tipiracil. Trifluridine is incorporated into DNA strand instead of thymidine in DNA synthesis process and inhibit progression of cell cycle. In placebo-controlled phase III, TAGS trial FTD/TPI improved overall survival of heavily pretreated metastatic gastric cancer compared with placebo (5.7 months; 95% CI 4.8–6.2 in the FTD/TPI group and 3.6 months; 95% CI 3.1–4.1 in the placebo group; hazard ratio 0.69; *p* = 0.00029) [31]. Survival benefit in later-line treatment had significant implication in gastric cancer, in which third-line treatment had not been established. Importantly, time to deterioration of ECOG performance status score (PS) to 2 or higher was statistically longer in the FTD/TPI group than in the placebo group (4.3 months vs 2.3 months [2∙0–2∙8] months; HR 0∙69; *p* = 0.00053). Although relatively high incidence of nausea and appetite loss in few days after drug induction and subsequent hematological toxicity in one to two weeks later, the data show that FTD/TPI is tolerable even in later lines of advanced gastric cancer. Recent evidence suggests that addition of anti-VEGF antibody, bevacizumab, improves the efficacy of FTD/TPI in colorectal cancer. Phase III, randomized controlled SUNLIGHT trial showed prolonged overall survival in FTD/ TPI plus bevacizumab group compared to FTD/TPI alone in refractory metastatic colorectal cancer [32]. However, in advanced gastric cancer, FTD/TPI plus bevacizumab did not improve PFS of previously treated advanced gastric cancer patients compared with FTD/TPI monotherapy [33]. Other anti-VEGF agent, ramucirumab, in combination with FTD/TPI demonstrated promising PFS and tumor response rate with the feasible safety profile [34]. Randomized phase II trial of FTD/TPI plus ramucirumab versus FTD/TPI alone, RETRIEVE study (WJOG15822G) is ongoing [35].

## Discussion

In later-line treatment, the tumor microenvironment undergoes significant alterations as a result of prior chemotherapy and molecular-targeted treatments. In this setting, considering the interactions between ICI, anti-VEGF therapy, and cytotoxic agents could be a crucial factor in the choice of subsequent treatment. The gastric cancer genome is characterized by focal amplification of oncogenes, including receptor tyrosine kinases, such as ERBB2 (HER2), EGFR, ERBB3, VEGF-A, KRAS/NRAS, MET, JAK2, and PD-L1/PD-L2 [36]. Among them, VEGF-A amplification is associated with neo-angiogenesis, and JAK2 and PD-L1/PD-L2 amplification leads to formation of the immunosuppressive tumor microenvironment. Hypoxia in pathological microenvironment accelerates the activation of VEGF signaling, which leads to increased infiltration of immunosuppressive cells, such as regulatory T cells (Tregs), tumor-associated macrophages (TAMs), especially those of the M2 phenotype, and myeloid-derived suppressor cells (MDSCs) [37]. These immune cells collectively suppress infiltration of CD8-positive T cells, playing a significant role in immunosuppression. In this regard, combination of anti-VEGF treatment with chemotherapy as well as ICIs holds significant importance in the treatment of gastric cancer. Recent data showed encouraging activity with new combination therapies including ramucirumab plus nivolumab or pembrolizumab, nivolumab plus regorafenib, and pembrolizumab plus lenvatinib [38].

The phase III trial evaluating the combination of ramucirumab with irinotecan did not demonstrate a significant prolongation of OS beyond progression for ramucirumab [29]. However, the significant improvements in ORR and PFS suggest potential benefits and warrant further exploration in the development of anti-VEGF combination therapy, such as FTD/TPI plus ramucirumab in later-line treatment of gastric cancer [35].

Gastric cancer, compared to lower gastrointestinal cancers, tends to result in sudden declines in performance status and quality of life due to aggressiveness of the disease. Molecular-targeted therapy, which shows relatively low-frequency of severe side effects, is expected to play a crucial role in gastric cancer treatment, particularly in cases where performance status is prone to deterioration. We hope that novel combination therapy including molecular-targeted therapies will reshape the landscape of late-line treatment for advanced gastric cancer.
